# Andexanet alpha versus four-factor prothrombin complex concentrate in DOACs anticoagulation reversal: an updated systematic review and meta-analysis

**DOI:** 10.1186/s13054-024-05014-x

**Published:** 2024-07-05

**Authors:** Daniele Orso, Federico Fonda, Alessandro Brussa, Irene Comisso, Elisabetta Auci, Marco Sartori, Tiziana Bove

**Affiliations:** 1 Azienda Sanitaria Universitaria Friuli Centrale, Department of Emergency “Santa Maria Della Misericordia”, University Hospital of Udine, Piazzale Santa Maria Della Misericordia, N.15, 33100 Udine, UD Italy; 2 Department of Medicine, University of Udine, via Colugna 50, 33100 Udine, Italy

**Keywords:** Andexanet alpha, 4F-PCC, Anticoagulation reversal, Haemorrhage, DOACs, Meta-analysis

## Abstract

**Background:**

There is currently a lack of evidence for the comparative effectiveness of Andexanet alpha and four-factor prothrombin complex concentrate (4F-PCC) in anticoagulation reversal of direct oral anticoagulants (DOACs). The primary aim of our systematic review was to verify which drug is more effective in reducing short-term all-cause mortality. The secondary aim was to determine which of the two reverting strategies is less affected by thromboembolic events.

**Methods:**

A systematic review and meta-analysis was performed.

**Results:**

Twenty-two studies were analysed in the systematic review and quantitative synthesis. In all-cause short-term mortality, Andexanet alpha showed a risk ratio (RR) of 0.71(95% CI 0.37–1.34) in RCTs and PSMs, compared to 4F-PCC (I^2^ = 81%). Considering the retrospective studies, the pooled RR resulted in 0.84 (95% CI 0.69–1.01) for the common effects model and 0.82 (95% CI 0.63–1.07) for the random effects model (*I*^2^ = 34.2%). Regarding the incidence of thromboembolic events, for RCTs and PSMs, the common and the random effects model exhibited a RR of 1.74 (95% CI 1.09–2.77), and 1.71 (95% CI 1.01–2.89), respectively, for Andexanet alpha compared to 4F-PCC (*I*^2^ = 0%). Considering the retrospective studies, the pooled RR resulted in 1.21 (95% CI 0.87–1.69) for the common effects model and 1.18 (95% CI 0.86–1.62) for the random effects model (*I*^2^ = 0%).

**Conclusion:**

Considering a large group of both retrospective and controlled studies, Andexanet alpha did not show a statistically significant advantage over 4F-PCC in terms of mortality. In the analysis of the controlled studies alone, Andexanet alpha is associated with an increased risk of thromboembolic events.

**Clinical trial registration:**

PROSPERO: International prospective register of systematic reviews, 2024, CRD42024548768.

**Supplementary Information:**

The online version contains supplementary material available at 10.1186/s13054-024-05014-x.

## Background

Direct oral anticoagulants (DOACs) are a class of drugs that act directly by inhibiting thrombin action or factor Xa, inhibiting both the intrinsic and the extrinsic pathways of the coagulation cascade. They possess advantages over the older vitamin K antagonists. In fact, their effects are more predictable than the latter and no laboratory monitoring is required. DOACs have been proven effective in treating thrombosis, such as deep venous thrombosis or pulmonary thromboembolism, as well as in preventing thromboembolic events in some pro-thrombotic conditions, such as atrial fibrillation [1, 2]. In case of haemorrhagic events, however, it becomes essential to counteract their anticoagulant effect effectively and quickly, without causing an increase in thromboembolic events. While the administration of vitamin K can reverse the effects of older vitamin K antagonists, there are several strategies to reverse the anticoagulant effect of DOACs. A monoclonal antibody (idarucizumab) has been developed to directly counteract the anticoagulant effects of dabigatran, which is a direct thrombin inhibitor [3]. For factor X inhibitors (such as rivaroxaban, apixaban, and edoxaban), the main reversal drug is the prothrombin complex (currently Four-factor prothrombin complex concentrate, i.e., 4F-PCC), which restores the level of molecules involved in the coagulation cascade [4]. Andexanet alpha is a modified recombinant inactive form of factor Xa that has been developed in recent years [5]. By binding and sequestering the molecules of the factor Xa inhibitor, it restores the thrombin generation mechanism. There is currently a lack of evidence for the comparative effectiveness of Andexanet alpha and 4F-PCC. A recently released randomized controlled trial, the ANNEXA-I trial raised further attention to the evidence gap, since results had shown that Andexanet alpha is more effective in limiting the expansion of hematoma in cases of intracerebral haemorrhage, but its use is associated with a greater incidence of thromboembolic events [6]. The scientific discussion on the topic was also fostered due to some intrinsic limitations of the study. In fact, it has been argued the outcome chosen as a measure of the effectiveness of the intervention is not patient-centred and therefore may be considered only indirectly relevant from the clinical point of view.

To fill the gap in the field with updated evidence, the primary aim of our systematic review was to verify which drug (Andexanet alpha or 4F-PCC) is more effective in reducing short-term all-cause mortality in anticoagulation reversal. We would verify this outcome both in cases of intracerebral haemorrhage (ICH) and non-intracerebral haemorrhage (such as gastrointestinal haemorrhage, traumatic haemorrhage, etc.). The secondary aim was to determine which of the two reverting strategies is less affected by thromboembolic events.

## Methods

A systematic review and meta-analysis of the literature was performed. The protocol of this review was prospectively registered in the International Prospective Register of Systematic Reviews (PROSPERO, CRD42024548768), and we reported this systematic review in accordance with the Preferred Reporting Items for Systematic Reviews and Meta-Analyses (PRISMA) Statement reporting guidelines [7].

### Eligibility criteria, search strategy and data collection

We considered any study that investigated the administration of Andexanet alpha or 4F-PCC to reverse an anticoagulation effect caused by a DOAC in cases of haemorrhage. Our search included randomized controlled trials (RCTs), observational prospective/retrospective studies, retrospective studies with propensity score matching and interventional studies. Qualitative studies, editorials, comments, letters to the editor, conference papers, case reports, clinical guidelines, or literature reviews with or without meta-analysis were excluded. Studies involving non-adult participants (i.e., < 18 years old), pregnant patients, animal subjects, and those that did not report outcome data were also excluded.

Searches were conducted using the electronic biomedical databases PubMed, Scopus, and Cumulative Index to Nursing and Allied Health Literature (CINHAL). To ensure a comprehensive synthesis of the available literature, existing meta-analyses on the same topic retrieved during the screening phase were retrieved and analysed to select relevant studies for inclusion. Search strings for each database were developed by one researcher (DO). Search strings were peer-reviewed prior to the execution [8] by an experienced researcher (FF) following the Peer Review of Electronic Search Strategies (PRESS) guidelines checklist [9]. Search results were imported into the Covidence platform by Veritas Health Innovation Ltd. The selection process consisted of two phases: title/abstract screening and full-text screening. After duplicate results were removed, the screening was performed independently and blindly by two researchers (TB and DO). When there were disagreements regarding article eligibility, a consensus was reached by rediscussing conflicting cases, and the final decision was made after discussion until a consensus was reached involving a third researcher (AB). The following data were extracted: name of the author(s), year, study design, sample size(s), the indication of reversal of anticoagulation (i.e., ICH or non-ICH), number of deaths in the intervention group and in the control group, thromboembolic events in the intervention group and in the control group. If available, the number of patients with a Rankin score > 3 in the intervention and control groups was reported. Any other variables reported in the studies were analysed and included when relevant to the systematic review's question. An electronic data extraction form was implemented using the Covidence platform and piloted with at least three of the articles selected to ensure its usefulness, appropriateness, and feasibility [8, 10]. The data was extracted cooperatively by two data extractors (DO and TB) who were previously trained and had the appropriate topic knowledge. Rediscussing conflicting cases led to a consensus for data extraction, and the final decision was made after the consensus was reached.

### Risk of bias assessment

The risk of bias (RoB) was assessed independently by two authors (DO and FF). For the controlled trials, the Revised Cochrane Risk-of-Bias Tool for Randomized Trials (RoB2) was used to assess the risk of bias [11]. In all remaining included studies, Robins-I (Risk Of Bias In Non-randomized Studies—of Interventions) was utilized [12]. In case of conflicting judgments, the authors discussed until they reached a consensus to resolve any disagreements.

### Statistical analysis

In the execution of the meta-analysis, a binary outcome (number of events for each of the two groups) was identified. Fixed-effect and random-effect analyses were conducted. The risk ratio was calculated using the Mantel–Haenszel method in the initial case. In the second case, the inverse-variance method was used. The *I*^*2*^ statistic was used to assess between-study inconsistency. The meta-analysis findings were presented using forest plots.

Our research focused on the 'outliers' to identify the potential causes of heterogeneity in studies. We conducted an Influence Analysis to determine the most influential cases that determine the heterogeneity between studies. The indication for anticoagulation reversal (ICH or non-ICH) was used to plan an analysis of the sample subgroups. The main features of the included studies were used to conduct a meta-regression to identify the possible causes of heterogeneity between studies. To evaluate publication bias, a Funnel Plot was utilized.

Analyses were performed using R version 4.3.2, using the packages meta, dmetar, tidyverse, metafor, ggplot2, gridExtra, and robvis.

## Results

### Study selection and characteristics

**Fig. 1:**
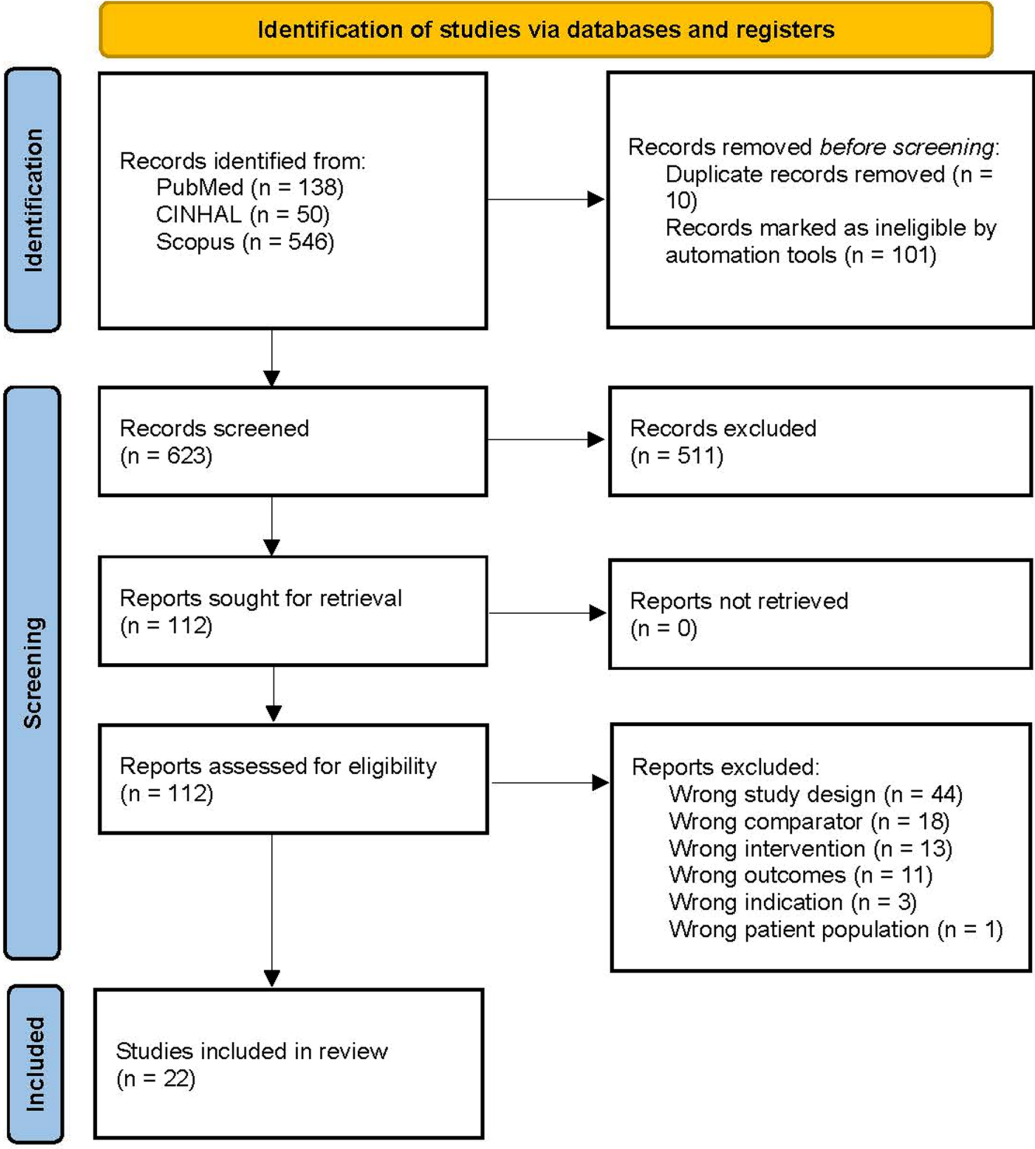
PRISMA flowchart

Six hundred twenty-three records were found during the initial identification process. We analysed 112 studies through full text after weeding out non-relevant and duplicate records (Fig. 1). Twenty-two studies were analysed in the systematic review and quantitative synthesis (Table 1).

**Table 1:** Characteristics of the studies included in the systematic review. Characteristics of the studies included in the systematic review. "Disability" refers to the number of patients with Rankin scores above 3. The brackets indicate the percentages relative to the total population

Study	Design	Indication	Population	AA Group (%)	4F-PCC Group (%)	Deads AA group (%)	Deads 4F-PCC group (%)	Disability AA group	Disability 4F-PCC group	Thromboembolic events AA group (%)	Thromboembolic events 4F-PCC group (%)
*Controlled studies*											
Connolly SJ 2024	RCT	ICH	530	263 (49.6)	267 (50.4)	73 (13.8)	68 (12.8)			27 (5.1)	15 (2.8)
Costa OS 2022	PSM	ICH	202	107 (53)	95 (47)	10 (5)	14 (6.9)			2 (1)	0 (0)
Parsels KA 2022	PSM	ICH	52	26 (50)	26 (50)					7 (13.5)	3 (5.8)
Keinath JJ 2023	PSM	non-ICH	340	170 (50)	170 (50)	28 (8.2)	23 (6.8)			9 (2.6)	8 (2.4)
Sutton SS 2023	PSM	non-ICH	255	85 (33.3)	170 (66.7)	9 (3.5)	43 (16.9)				
Cohen AT 2021	PSM	mixed	410	322 (78.5)	88 (21.5)	47 (11.5)	30 (7.3)				
*Retrospective studies*											
Barra ME 2020	Retrospective	ICH	29	18 (62.1)	11 (37.9)	4 (13.8)	7 (24.1)	10 (34.5)	1 (3.4)	3 (10.3)	1 (3.4)
Pham H 2022	Retrospective	ICH	109	47 (43.1)	62 (56.9)	16 (14.7)	13 (11.9)	9 (8.3)	5 (4.6)	4 (3.7)	6 (5.5)
Siepen BM 2024	Retrospective	ICH	243	180 (74.1)	63 (25.9)	11 (4.5)	12 (4.9)			20 (8.2)	6 (2.5)
Schmidt LE 2022	Retrospective	non-ICH	85	33 (38.8)	52 (61.2)	6 (7.1)	9 (10.6)			6 (7.1)	2 (2.4)
Oh ES 2023	Retrospective	ICH	24	9 (37.5)	15 (62.5)	0 (0)	2 (8.3)				
Troyer C 2023	Retrospective	ICH	46	31 (67.4)	15 (32.6)	4 (8.7)	5 (10.9)			2 (4.3)	0 (0)
Vestal ML 2022	Retrospective	ICH	56	21 (37.5)	35 (62.5)	6 (10.7)	14 (25)			3 (5.4)	11 (19.6)
Stevens VM 2021	Retrospective	non-ICH	32	16 (50)	16 (50)	2 (6.25)	5 (15.6)			4 (12.5)	3 (9.4)
Sadek E 2024	Retrospective	non-ICH	324	59 (18.2)	265 (81.8)	15 (4.6)	49 (15.1)				
Irizarry-Gatell VM	Retrospective	ICH	89	23 (25.8)	66 (74.2)	5 (5.6)	17 (19.1)				
Lipski M 2022	Retrospective	ICH	70	23 (32.9)	47 (67.1)	7 (10)	13 (18.6)			5 (7.1)	8 (11.4)
Singer AJ 2023	Retrospective	mixed	100	50 (50)	50 (50)	8 (8)	9 (9)			7 (7)	8 (8)
Huttner HB 2022	Retrospective	ICH	182	85 (46.7)	97 (53.3)	14 (7.7)	20 (11)	44 (24.2)	67 (36.8)	11 (6)	8 (4.4)
Ammar AA 2021	Retrospective	ICH	44	28 (63.6)	16 (36.4)	11 (25)	6 (13.6)			2 (4.5)	0 (0)
Milioglou I 2022	Retrospective	ICH	45	23 (51.1)	22 (48.9)	11 (24.4)	10 (22.2)				
Koo SJ 2024	Retrospective	non-ICH	183	84 (45.9)	99 (54.1)	10 (5.5)	20 (10.9)			6 (3.3)	7 (3.8)

Characteristics of the studies included in the systematic review. “Disability” refers to the number of patients with Rankin scores above 3. The brackets indicate the percentages relative to the total population

AA, Andexanet alpha; ICH, intracranial haemorrhage; RCT, randomized controlled trial; 4F-PCC, Four-factor prothrombin complex concentrate

Six studies were RCTs or retrospective studies in which the authors prepared some form of attenuation of the imbalance between the characteristics of the two groups (i.e., Propensity Score Matching, PSMs) [6, 13–17]. Three of these studies included patients with ICH [6, 13, 14]. Two studies considered patients with haemorrhages other than ICH [15, 16] and one considered both types of haemorrhagic events [17]. Only 4 studies reported the incidence of thrombotic events in the two groups of patients [6, 13–15]. The rate of patients with Rankin score was not reported in any of these studies [6, 13–17].

Sixteen studies were retrospective [18–33]. Eleven studies included patients with ICH [18–20, 22–24, 27, 28, 30–32], while four studies included patients with non-ICH [21, 25, 26, 33], and one study included a mixed population [29]. Twelve studies reported the thrombotic events rate alongside the mortality rate. Only three studies reported the rate of patients with Rankin scores [18, 19, 30].

### Risk of bias

**Fig. 2:**
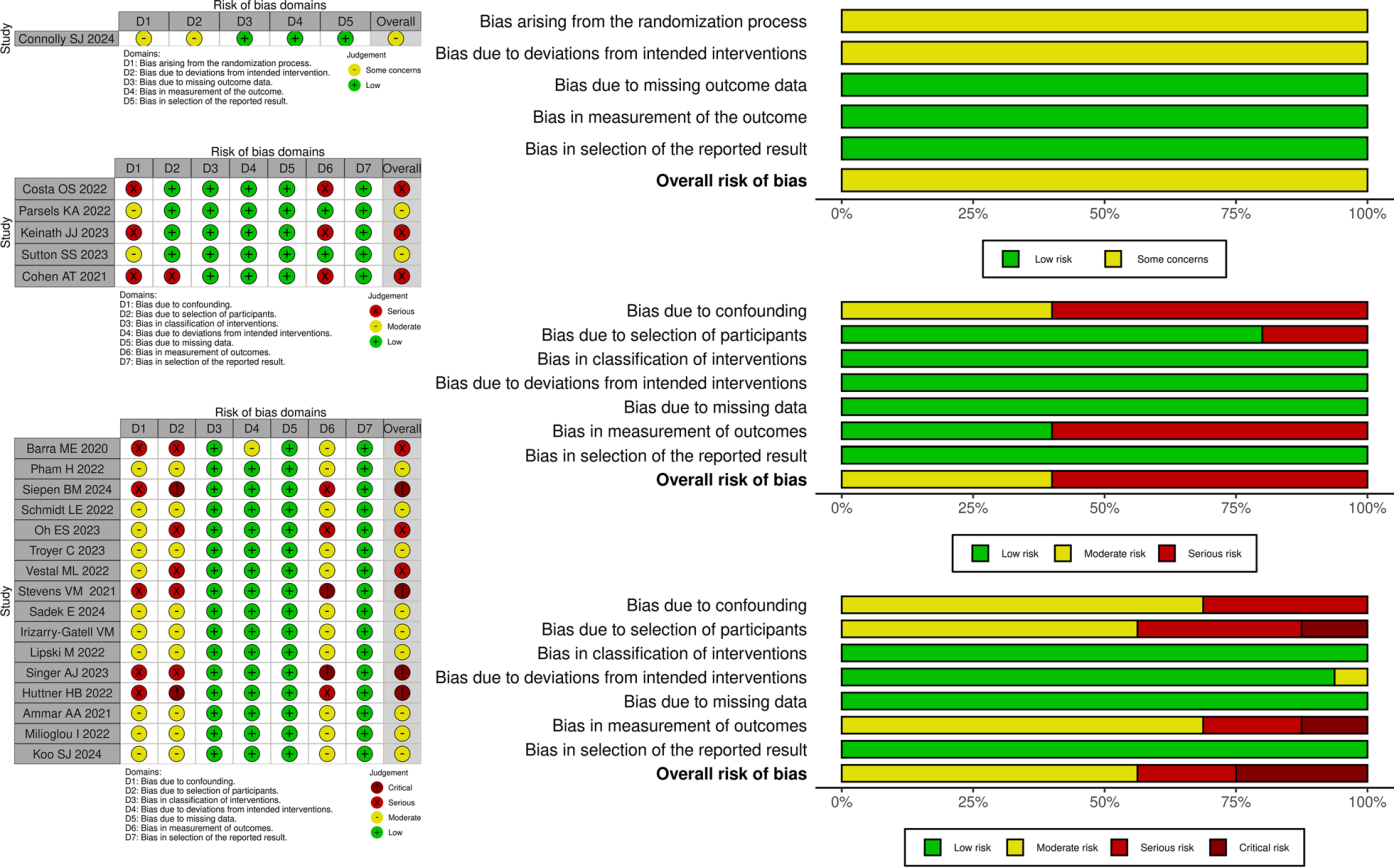
Risk of bias of the included studies. For the controlled trials, the RoB2 was used to assess the risk of bias. In all remaining included studies, Robins-I was utilized. Patient selection was the primary cause of bias in the studies that were included. For further details see the main text

Patient selection was the primary cause of bias in the studies that were included (Fig. 2). Retrospective studies were more susceptible to bias. Several studies have grouped and compared two distinct populations [20, 25, 29, 30]. The populations do not appear to be fully comparable, either because they were from different and independent previous trials or because the two groups in the study were enrolled at different times. The use of this methodological selection exposes these studies to a critical risk of bias. The inclusion of two study groups in a non-consecutive or sequential manner made the implicated studies more likely to influence researchers' awareness of the outcome for one of the two drugs studied. The controlled studies also raised concerns about the actual balance of the two study groups.

### Quantitative synthesis for all-cause short-term mortality

**Fig. 3:**
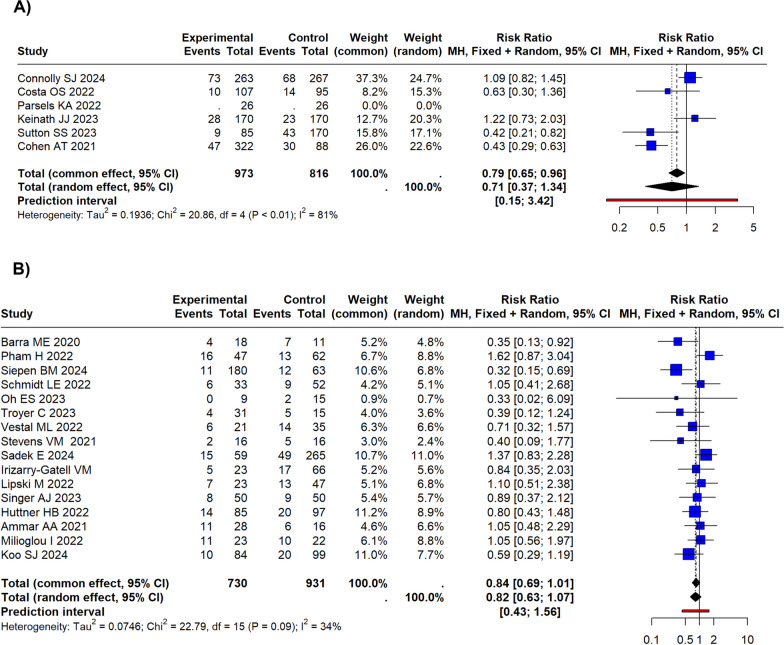
Forest plots for all-cause short-term mortality. **A** Forest plot for the controlled (RCT and PSM) studies. The random effects model exhibited a risk ratio (RR) of 0.71 (95% CI 0.37–1.34). Since the confidence interval crosses the unit, the difference was not statistically significant. **B** Forest plot for the retrospective studies. The pooled RR resulted in 0.84 (95% CI 0.69–1.01) for the common effects model and 0.82 (95% CI 0.63–1.07) for the random effects model

The random effects model showed a risk ratio (RR) of 0.71 (95% CI 0.37–1.34) for Andexanet alpha group compared to 4F-PCC (as reference) in RCTs and PSMSs, as shown in Fig. 3. Since the confidence interval crosses the unit, the difference was not statistically significant. By subdividing the population based on the indication to anticoagulation reversal, the studies about ICH showed a RR of 0.94 (95% CI 0.04–20.40; *I*^2^ = 41.2%), the non-ICH studies showed a RR of 0.73 (95% CI 0.00–640.25; *I*^2^ = 83.8%), and the mixed population study showed a RR of 0.43 (95% CI 0.29–0.63) (Fig. 3S in Supplemental Material).

**Fig. 4:**
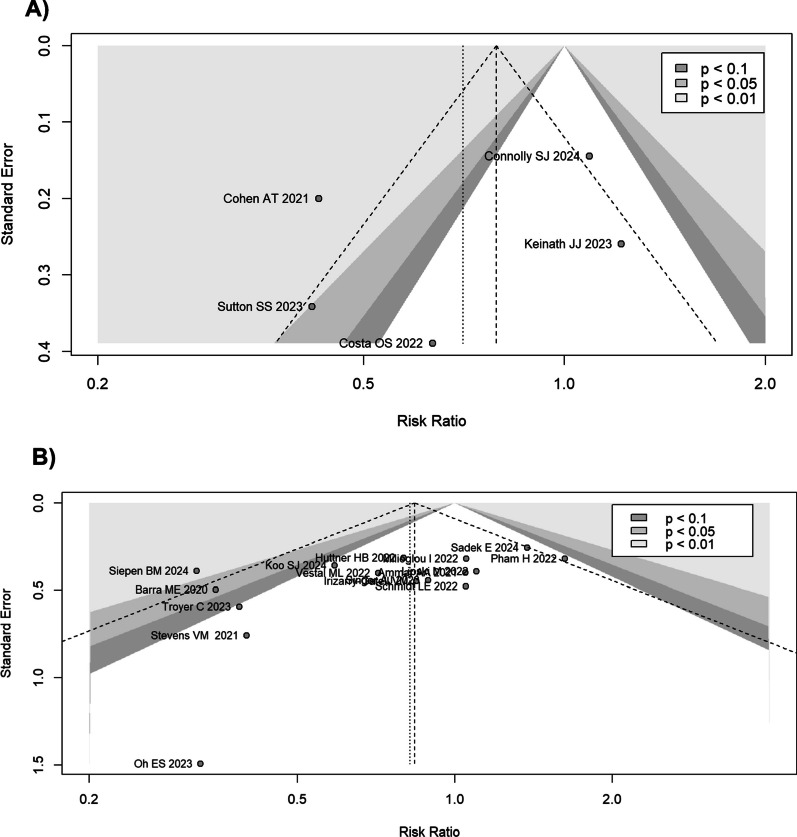
Funnel plot for all-cause short-term mortality. **A** Funnel plot for the controlled (RCT and PSM) studies. Egger’s test was not statistically significant (*p* = 0.47). **B** Funnel plot for the retrospective studies. Egger’s test was statistically significant (*p* = 0.03). Some studies show an excess reduction of mortality in favour of the Andexanet alpha group

In the pooled model, the *I*^2^ was 81%. The study by Cohen et al. appeared to be particularly distant from the results of the other studies. When this study was removed, the model showed that the Andexanet alpha group had an RR of 0.82 (95% CI 0.38–1.79; I^2^ = 65.3%). According to meta-regression analysis, the study design type led to around 8% of heterogeneity (*R*^2^ = 7.83%). The funnel plot did not show a significant publication bias (Egger’s test *p* = 0.47) (Fig. 4).

Considering the retrospective studies analysing Andexanet alpha versus 4F-PCC groups, the pooled RR resulted in 0.84 (95% CI 0.69–1.01) for the common effects model and 0.82 (95% CI 0.63–1.07) for the random effects model (Fig. 3). The pooled *I*^2^ was 34.2%. By subdividing the population based on the indication for anticoagulation reversal, the studies on Andexanet alpha versus 4F-PCC in ICH patients showed a RR of 0.79 (95% CI 0.63–1.00, *I*^2^ = 41.0%) (Fig. 4S in Supplemental Material). The non-ICH studies showed a RR of 0.93 (95% CI 0.65–1.32, *I*^2^ = 41.9%), and the mixed population study showed a RR of 0.89 (95% CI 0.37–2.12).

The study that differed most from the results of the other studies was that by Siepen et al. However, excluding it, the RR did not change substantially (RR 0.90; 95% CI 0.74–1.10 for the common effects model; RR 0.91; 95% CI 0.71–1.15 for the random effects model), although a reduction of the between-studies inconsistency was achieved (*I*^2^ = 12.4%). At meta-regression analysis, the different indications for anticoagulation reversal were  not significantly responsible for a residual heterogeneity (*R*^2^ = 0%). The funnel plot showed a significant publication bias (Egger’s test *p* = 0.03) (Fig. 4).

### Quantitative synthesis for the thromboembolic events

**Fig. 5:**
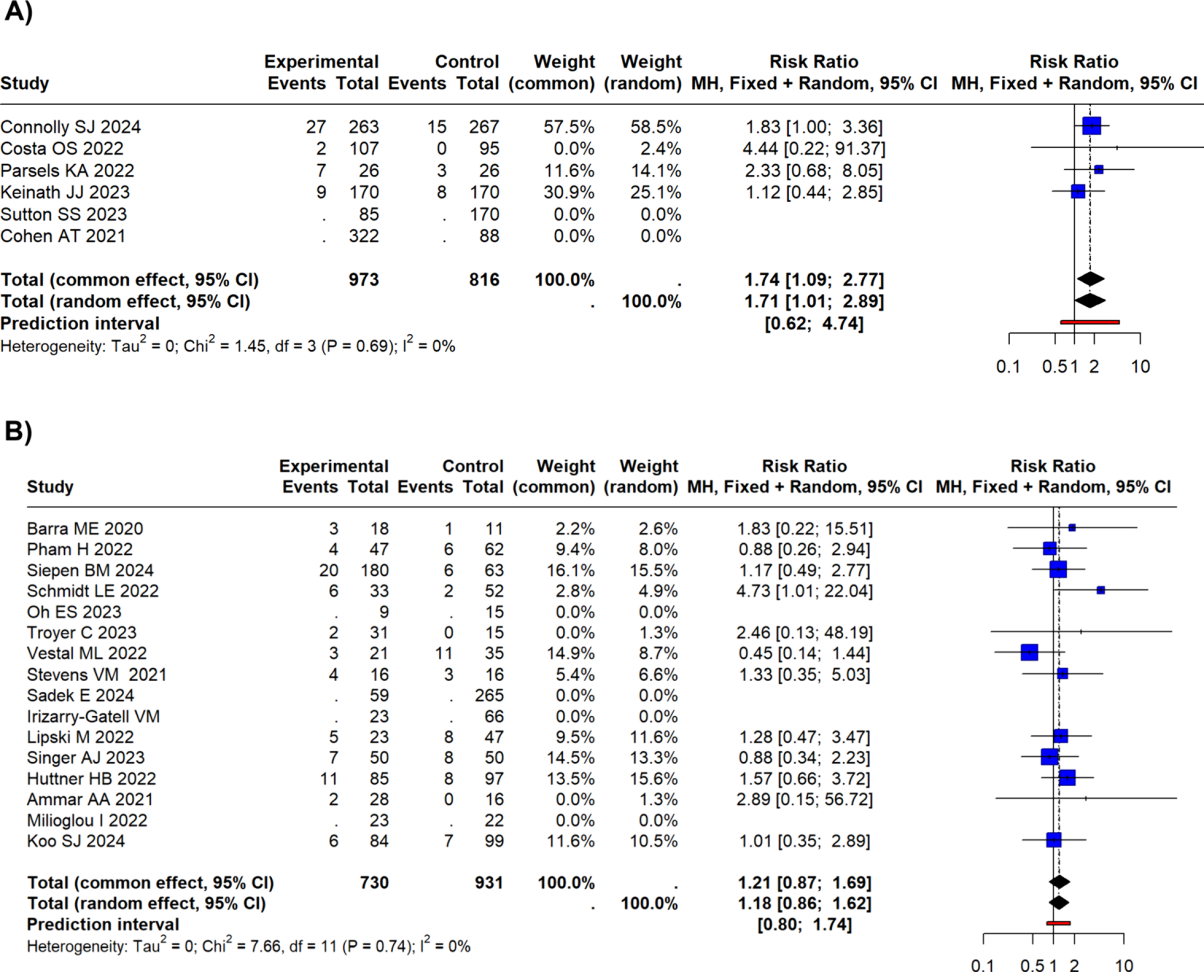
Forest plots for thromboembolic events. **A** For RCTs and PSMs, the common and the random effects model exhibited a RR of 1.74 (95% CI 1.09–2.77], and 1.71 (95% CI 1.01–2.89), respectively. **B** Forest plot for the retrospective studies. The pooled RR resulted in 0.84 (95% CI 0.69–1.01) for the common effects model and 0.82 (95% CI 0.63–1.07) for the random effects model. **B** Considering the retrospective studies, the pooled RR resulted in 1.21 (95% CI 0.87–1.69) for the common effects model and 1.18 (95% CI 0.86–1.62) for the random effects model

For RCTs and PSMSs, the common and random effects models respectively showed an RR of 1.74 (95% CI 1.09–2.77) and 1.71 (95% CI 1.01–2.89) for Andexanet alpha compared to 4F-PCC (Fig. 5). The pooled *I*^2^ was 0.

**Fig. 6:**
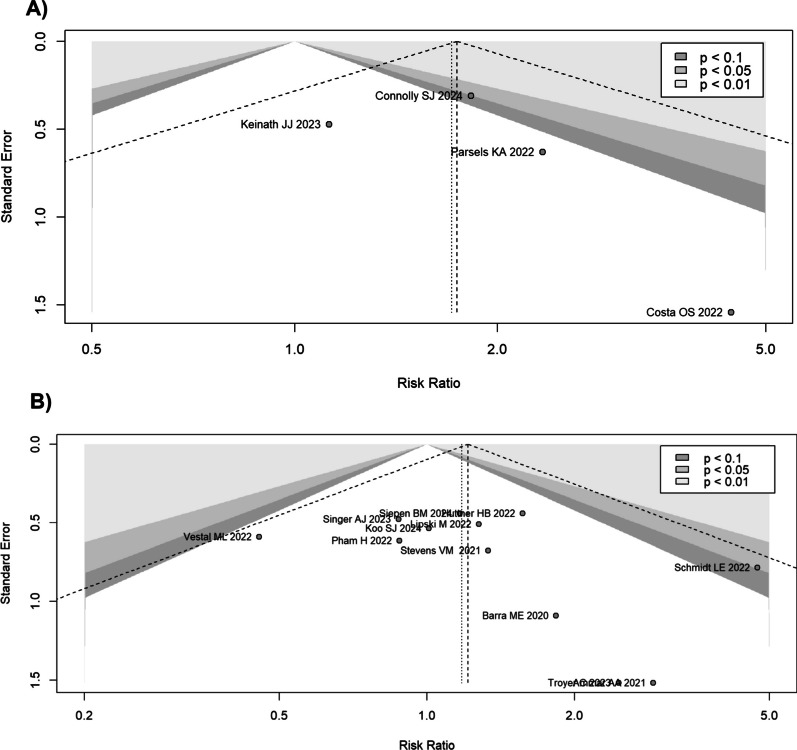
Funnel plot for thromboembolic events. **A** Funnel plot for the controlled (RCT and PSM) studies. Egger’s test was not statistically significant (*p* = 0.59). **B** Funnel plot for the retrospective studies. Egger’s test was not statistically significant (*p* = 0.22)

By subdividing the population based on the indication for anticoagulation reversal, the studies on the ICH population showed a RR for Andexanet alpha of 2.02 (95% CI 1.17–3.47) for the common effects model and 1.97 (95% CI 1.16–3.35); *I*^2^ = 41.2%) for the random effects model. In the non-ICH population studies, Andexanet alpha was found to have an RR of 1.13 (0.44–2.85) for the common effects model and 1.13 (0.44–2.85) for the random effects model compared to 4F-PCC (Fig. 7S in Supplemental Material). The funnel plot did not show a significant publication bias (Egger’s test *p* = 0.59) (Fig. 6).

Considering the retrospective studies, the pooled RR resulted in 1.21 (95% CI 0.87–1.69) for the common effects model and 1.18 (95% CI 0.86–1.62) for the random effects model for Andexanet alpha group vs 4F-PCC group (Fig. 5). The pooled *I*^2^ was 0.

By subdividing the population based on the indication for anticoagulation reversal, the studies about the ICH population showed a RR of 1.16 (95% CI 0.76–1.77, *I*^2^ = 0%) for the common effects model and 1.14 (95% CI 0.77–1.68) for Andexanet group (Fig. 8S in Supplemental Material). The non-ICH population studies showed a RR of 1.62 (95% CI 0.81- 3.25; *I*^2^ = 26%) for the common effects model, and 1.62 (95% CI 0.24–11.07) for the random effects model; for the mixed population study the RR was 0.88 (95% CI 0.34–2.23) for both the models. The funnel plot did not show a significant publication bias (Egger’s test *p* = 0.22) (Fig. 6).

## Discussion

This review provides a summary of the evidence on the effectiveness of Andexanet alpha compared to 4F-PCC for short-term all-cause mortality. We found no statistically significant difference in the two comparison groups either in the controlled studies (RCTs and PSMs) or in the retrospective studies, either in the case of ICH or in the case of non-ICH. In this respect, while the high between-studies inconsistency makes the conclusions of the controlled studies less reliable, the low inconsistency of the pooled retrospective studies supports this conclusion. Regarding the incidence of thromboembolic events, the analysis of controlled studies shows an increase in relative risk in the Andexanet alpha group compared to the 4F-PCC group. This effect seems to be particularly significant for ICH-population studies. This conclusion is not confirmed by the analysis of retrospective studies, for which there is no different incidence of thromboembolic events between the two groups.

The rate of ICH due to factor Xa inhibitors is believed to be about one in 500–1000 per patient-year [34] and the rate of non-intracranial haemorrhages is near 19 per 100 patient-year [35]. Although DOACs seem at least as safe as the old Vitamin K antagonists in terms of the incidence of ICH (7% vs 11%) [36], evidence for the best reversal strategy for the anticoagulant effect is still lacking. Recently, the first (and so far, unique) RCT that directly compares Andexanet alpha to the usual therapy (i.e., 4F-PCC) was published, called ANNEXA-I [6]. The expectation of the results of this study was proportional to the controversies arising from its publication. The main criticisms were the primary outcome and the high patient rate in the control group without any treatment. The primary outcome of the trial was the expansion of intracerebral hematoma less than 35% of the volume at 12 h after administration of the drug. There was a statistically significant difference for the group of patients who were given Andexanet alpha (67.0% vs 53.1%) in this regard. However, the 30-day mortality rate was not statistically significant between the two groups. This result was achieved even though approximately 15% of patients in the control group did not receive any treatment (the so-called “passive reversal” strategy). In addition, the intervention group showed a higher rate of thromboembolic events (i.e., myocardial infarction, ischemic stroke, etc.) than the control group (10.3% vs 5.6%). Our meta-analysis confirms that there are no significant differences in short-term all-cause mortality between the two groups, which is consistent with the results of most of the studies (both controlled and retrospective). However, the ANNEXA-I study, like most of the studies included in this meta-analysis, was not designed to address mortality as a primary outcome. In fact, mortality is influenced by the location of bleeding, the patient's clinical condition, as well as the extent of bleeding [37]. In addition, ANNEXA-I is the only trial that has set inclusion criteria that exclude GCS score < 7 or NHISS score > 35, as well as scheduled surgery less than 12 h. Due to their retrospective nature, other studies on ICH patients do not have any specific exclusion criteria for severity of bleeding. However, ANNEXA-I did not demonstrate any significant advantage in using Andexanet-alpha over 4F-PCC in terms of short-term mortality. Although in this study patients were theoretically less severe than other studies, the overall mortality rate in the ANNEXA-I study was not significantly different from other studies on patients with ICH (23% for ANNEXA-I vs 27%, median value in the other studies). In this respect, the mortality rate was homogeneous (i.e., around 20%) for studies on non-ICH patients, while in studies on ICH population, the mortality rate varied from 8%, as reported by Oh et al. to 47% of Milioglou et al. (Table 2S in Supplemental Material).

Regarding disability, we found that most studies did not report this outcome or reported it in a non-standardized manner. This limitation prevents the possibility of evaluating the effectiveness of anticoagulation reversal based on this patient-centred outcome.

Our meta-analysis differs significantly from previous ones in terms of the risk of thromboembolic events in the Andexanet alpha group compared to the 4F-PCC group. In fact, we found a statistically significant higher incidence of thromboembolic events in the group receiving Andexanet alpha than in the 4F-PCC group (for controlled studies but not for retrospective studies). The data analysis shows that the ANNEXA-I trial has the most significant impact. In fact, this study has a weight of almost 60%. Most studies included in our meta-analysis were not published at the time of the previous meta-analyses [38–40].  For example, most studies in our meta-analysis were not included by Shrestha et al. and by da Luz et al. because they were not published during their meta-analyses [38, 39]. Compared to the meta-analysis of Chaudhary et al., our meta-analysis also includes studies that have enrolled patients with extra-cranial bleeding [41]. However, the main difference is related to the inclusion of the ANNEXA-I trial which, as already highlighted, drags the result of the meta-analysis regarding the safety outcome.

The effectiveness of Andexanet alpha in thrombin generation was found to be higher than 4F-PCC in a recent ex vivo study, but no significant difference was observed in the remaining haemostatic reversal tests [42]. The restoration of coagulative cascade factors is confirmed by previous in vitro studies, with Andexanet alpha showing greater thrombin restoring ability at low DOACs concentrations [43, 44]. However, regarding the main outcome of this meta-analysis, namely mortality, Andexanet alpha does not appear to be more effective than the current standard reference (i.e., 4F-PCC). The relevance of this result is based on the higher cost of Andexanet alpha compared to 4F-PCC. Due to the increased cost in comparison to the current 4F-PCC strategy, certain clinicians may be seeking an advantage to achieve strong patient-centred outcomes [45]. In addition, concerns about safety in terms of increased incidence of thromboembolic events—although demonstrated only in patients with ICH (but by the only RCT published so far)—are to be considered carefully and deserve to be investigated with additional RCTs.

### Limitations

The main limitation of our meta-analysis lies in the impossibility of establishing disability (or functional recovery) as an outcome, which may be considered one of the most important (if not the most important at all) in terms of patient-centred outcomes research. This limitation is due to the low frequency with which studies explicitly or standardize this data. This is crucial since mortality, despite being another patient-centred outcome, is only indirectly linked to the use of a single drug and, instead, is due to various causal factors.

The nearest mortality was only considered because it has a more significant correlation with the haemorrhagic event than the 30-day mortality, which is not in line with previous meta-analyses' choices [40]. It must be noted that in certain studies, such as the nearest mortality study, 30-day mortality was taken into consideration.

Further limitation is the large rate of inconsistency between studies that we found in the primary outcome analysis of controlled studies. This, as already mentioned, is linked to the different types and qualities of the aggregate studies. Our analysis reveals that the quality of any of the studies considered is generally not optimal, especially for the selection and imbalance of the two groups of patients. Therefore, RCTs that are both high-quality and methodologically correct are needed.

## Conclusion

Considering a large group of both retrospective and controlled studies, Andexanet alpha did not show a statistically significant advantage over 4F-PCC in terms of mortality. In the analysis of the controlled studies alone, Andexanet alpha is associated with an increased risk of thromboembolic events.

### Supplementary Information

Additional file1 (PDF 738 KB)

## Data Availability

The data that support the findings of this study are available from the corresponding author upon reasonable request.

## Abbreviations

DOACs: Direct oral anticoagulants
4F-PCC: Four-factor prothrombin complex concentrate
ICH: Intracerebral haemorrhage
PROSPERO: International prospective register of systematic reviews
PRISMA: Preferred reporting items for systematic reviews and meta-analyses
RCT: Randomized controlled trial(s)
PRESS: Peer review of electronic search strategies
RoB: Risk of bias
RoB2: Revised Cochrane risk-of-bias tool for randomized trials
Robins-I: Risk of bias in non-randomized studies of interventions
PSM: Propensity score matching
RR: Risk ratio
95% CI: 95% Confidence interval
