# Supplementary material for: Andexanet alpha versus four-factor prothrombin complex concentrate in DOACs anticoagulation reversal: an updated systematic review and meta-analysis
Source: Crit Care. 2024 Jul 5;28:221. doi: 10.1186/s13054-024-05014-x (PMC11225147; doi:10.1186/s13054-024-05014-x)
Supplement: Supplementary file 1 — Additional file1 (PDF 738 KB) [file 13054_2024_5014_MOESM1_ESM.pdf]

Supplementary Material

Table 1s. Search strategy

Figure 1s. Baujat plots for mortality. Top, controlled studies; bottom, retrospective studies. On the x-axis the contribution to the overall heterogeneity is placed; on the y-axis the contribution on the overall effect size is placed.

Figure 2s. Influence plot for mortality. Top, controlled studies; bottom, retrospective studies.

Figure 3s. Forest plot for RCTs/PSMS on all-cause short-term mortality for the subpopulations based on indication of DOACs reversal (ICH vs. non-ICH)

Forest 4s. Forest plot for retrospective studies on all-cause short-term mortality for subpopulations based on indication for DOACs reversal (ICH vs. non-ICH)

Figure 5s. Baujat plots for thromboembolic events. Top, controlled studies; bottom, retrospective studies. On the x-axis the contribution to the overall heterogeneity is placed; on the y-axis the contribution on the overall effect size is placed.

Figure 6s. Influence plot for thromboembolic events. Top, controlled studies; bottom, retrospective studies.

Figure 7s. Forest plots for RCTs/PSMS in subpopulations that are identified by indication of DOACs reversal (ICH vs. non-ICH)

Figure 8s. Forest plot for retrospective studies on thromboembolic events among subpopulations based on the indication for DOAcs reversal (ICH vs. non-ICH)

Table 2s. Mortality rate in the included studies. RCT: Randomized Controlled Trial; PSM: Propensity Score Matching.

Library	Query	Records
PubMed	(“andexanet” [Mesh] OR “andexanet” [tiab] OR “DOACs-reversal” [tiab] OR “anticoagulation reversal” [tiab])<br>AND<br>(“PCC4” [Mesh] OR “PCC4”[tiab] OR “prothrombin complex”[tiab] OR “Prothrombin complex concentrate” [Mesh] OR “prothrombin complex concentrate” [tiab])<br>AND<br>(“outcom*” [tiab] OR “Critical Care Outcomes” [Mesh] OR “critical care outcomes” [tiab] OR “Patient Outcome Assessment” [Mesh] OR “patient outcome assessment” [tiab] OR “Treatment Outcome” [Mesh] OR “treatment outcome” [tiab] OR “Mortality” [Mesh] OR “mortality”[tiab] OR “Hospital Mortality” [Mesh] OR “hospital mortality” [tiab])	138
Scopus	(INDEXTERMS(“andexanet”) OR TITLE-ABS(“andexanet”) OR TITLE-ABS (“DOACs-reversal”) OR TITLE-ABS(“anticoagulation reversal”))<br>AND<br>(INDEXTERMS(PCC4) OR TITLE-ABS(PCC4) OR TITLE-ABS(“DOACs”)<br>AND<br>(TITLE-ABS(outcom*) OR INDEXTERMS(“Outcomes”) OR TITLE-ABS(“outcomes”) OR INDEXTERMS(“Patient Outcome Assessment”) OR TITLE-ABS(“patient outcome assessment”) OR INDEXTERMS(“Treatment Outcome”) OR TITLE-	546

	ABS("treatment outcome") OR INDEXTERMS(Mortality) OR TITLE-ABS(mortality) OR INDEXTERMS("Hospital Mortality") OR TITLE-ABS("hospital mortality"))	
CINHAL	((MH "andexanet") OR (TI "andexanet"; OR AB "andexanet") OR (TI "DOACs-reversal" OR AB "DOACs-reversal") OR (TI "anticoagulation reversal" OR AB "anticoagulation reversal")<br>AND<br>((MH "PCC4") OR (TI "PCC4"; OR AB "PCC4") OR (TI "prothrombin complex" OR AB "prothrombin complex") OR (TI "Prothrombin complex concentrate" OR AB "Prothrombin complex concentrate")<br>AND<br>((TI outcom* OR AB outcom*) OR (MH " Outcomes" OR (TI "outcomes" OR AB "outcomes") OR (MH "Patient Outcome Assessment+") OR (TI "patient outcome assessment"; OR AB "patient outcome assessment") OR (MH "Treatment Outcome+") OR (TI "treatment outcome" OR AB "treatment outcome") OR (MH Mortality+) OR (TI mortality OR AB mortality) OR (MH "Hospital Mortality+") OR (TI "hospital mortality" OR AB "hospital mortality"))	50

Table 1s.

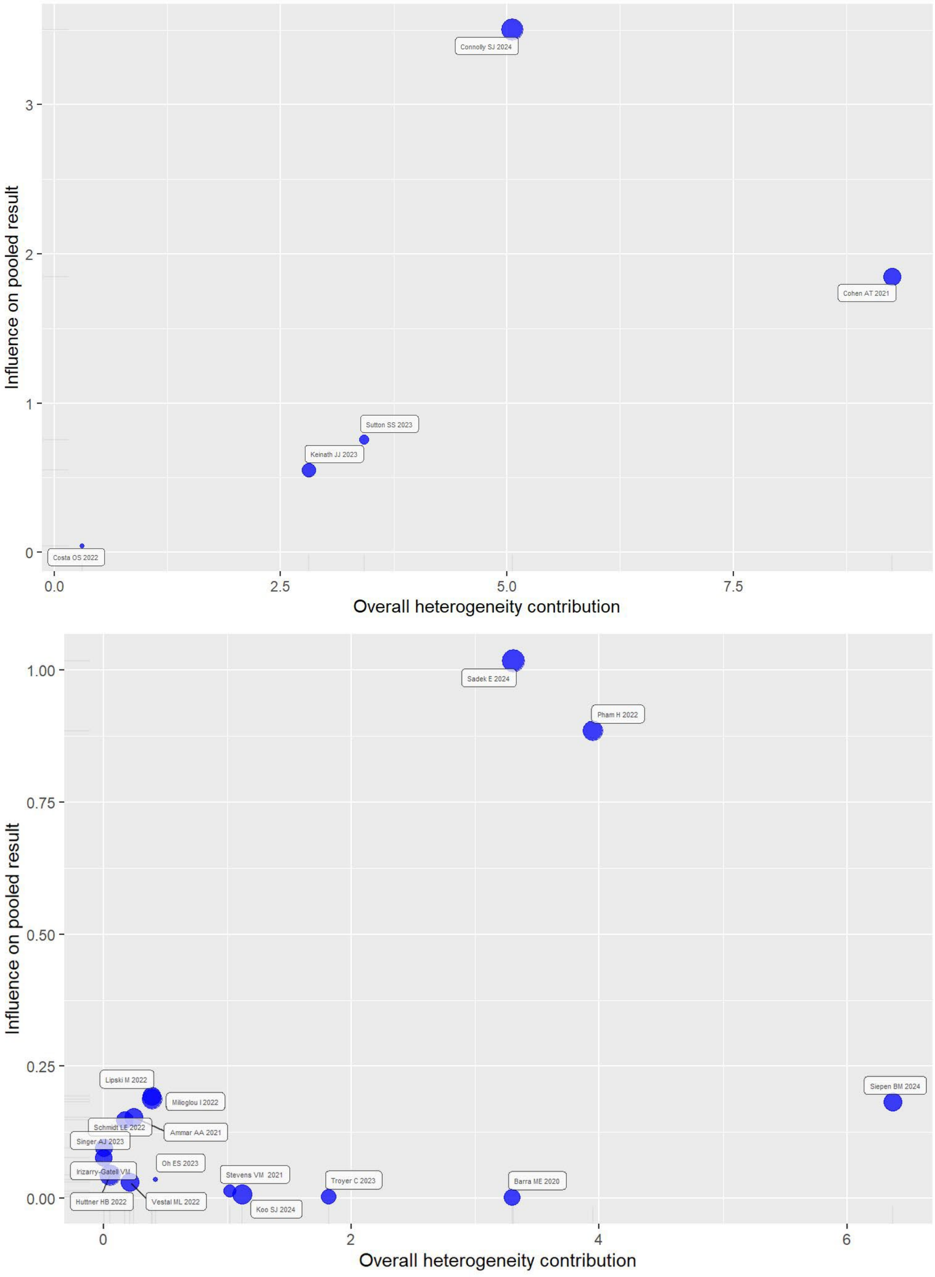
Connolly SJ 2024
Cohen AT 2021
Sutton SS 2023
Keinath JJ 2023
Costa OS 2022
Influence on pooled result
Overall heterogeneity contribution
0
1
2
3
0.0
2.5
5.0
7.5
Sadek E 2024
Pham H 2022
Siepen BM 2024
Lipski M 2022
Milioglou I 2022
Schmidt LE 2022
Ammar AA 2021
Singer AJ 2023
Oh ES 2023
Irizarry-Gatell VM
Stevens VM 2021
Troyer C 2023
Barra ME 2020
Huttner HB 2022
Vestal ML 2022
Koo SJ 2024
Influence on pooled result
Overall heterogeneity contribution
0.00
0.25
0.50
0.75
1.00
0
2
4
6

Figure 1s.

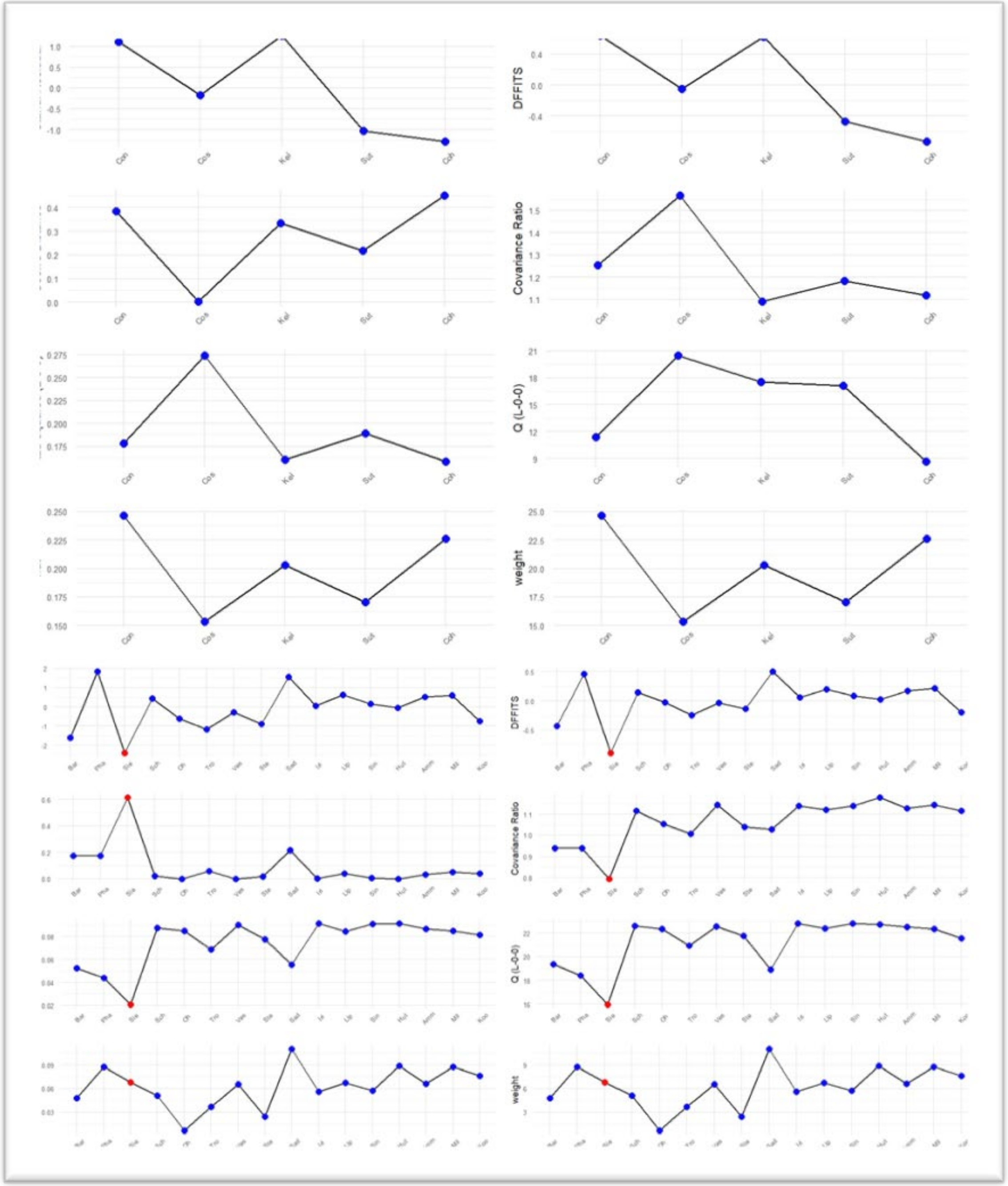

Figure 2s.

Study	Experimental Events	Experimental Total	Control Events	Control Total	RR	95%-CI	Weight (common)	Weight (random)
**Indication = ICH**								
Connolly SJ 2024	73	263	68	267	1.09	[0.82; 1.45]	37.3%	24.7%
Costa OS 2022	10	107	14	95	0.63	[0.30; 1.36]	8.2%	15.3%
Parsels KA 2022	.	26	.	26			0.0%	0.0%
**Common effect model**		**396**		**388**	**1.01**	**[0.77; 1.31]**	**45.5%**	**.**
**Random effects model**					**0.94**	**[0.04; 20.40]**	**.**	**40.0%**
Heterogeneity: $I^2 = 41\%$, $\tau^2 = 0.0604$, $p = 0.19$								
**Indication = non-ICH**								
Keinath JJ 2023	28	170	23	170	1.22	[0.73; 2.03]	12.7%	20.3%
Sutton SS 2023	9	85	43	170	0.42	[0.21; 0.82]	15.8%	17.1%
**Common effect model**		**255**		**340**	**0.77**	**[0.52; 1.15]**	**28.5%**	**.**
**Random effects model**					**0.73**	**[0.00; 640.25]**	**.**	**37.3%**
Heterogeneity: $I^2 = 84\%$, $\tau^2 = 0.4777$, $p = 0.01$								
**Indication = mixed**								
Cohen AT 2021	47	322	30	88	0.43	[0.29; 0.63]	26.0%	22.6%
**Common effect model**		**973**		**816**	**0.79**	**[0.65; 0.96]**	**100.0%**	**.**
**Random effects model**					**0.71**	**[0.37; 1.34]**	**.**	**100.0%**

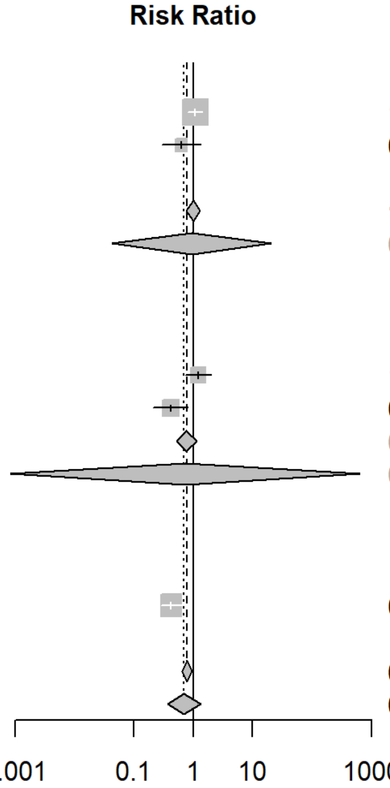

Heterogeneity: $I^2 = 81\%$, $\tau^2 = 0.1936$, $p < 0.01$

Test for subgroup differences (common effect): $\chi^2_2 = 12.55$, df = 2 ($p < 0.01$)

Test for subgroup differences (random effects): $\chi^2_2 = 6.37$, df = 2 ($p = 0.04$)

Figure 3s.

Study	Experimental Events	Experimental Total	Control Events	Control Total	RR	95%-CI	Weight (common)	Weight (random)
**Indication = ICH**								
Barra ME 2020	4	18	7	11	0.35	[0.13; 0.92]	5.2%	4.8%
Pham H 2022	16	47	13	62	1.62	[0.87; 3.04]	6.7%	8.8%
Siepen BM 2024	11	180	12	63	0.32	[0.15; 0.69]	10.6%	6.8%
Oh ES 2023	0	9	2	15	0.33	[0.02; 6.09]	0.9%	0.7%
Troyer C 2023	4	31	5	15	0.39	[0.12; 1.24]	4.0%	3.6%
Vestal ML 2022	6	21	14	35	0.71	[0.32; 1.57]	6.3%	6.6%
Irizarry-Gatell VM	5	23	17	66	0.84	[0.35; 2.03]	5.2%	5.6%
Lipski M 2022	7	23	13	47	1.10	[0.51; 2.38]	5.1%	6.8%
Huttner HB 2022	14	85	20	97	0.80	[0.43; 1.48]	11.2%	8.9%
Ammar AA 2021	11	28	6	16	1.05	[0.48; 2.29]	4.6%	6.6%
Milioglou I 2022	11	23	10	22	1.05	[0.56; 1.97]	6.1%	8.8%
**Common effect model**		488		449	0.79	[0.63; 1.00]	65.9%	.
**Random effects model**					0.78	[0.54; 1.11]	.	68.1%
Heterogeneity: $I^2 = 41\%$, $\tau^2 = 0.1095$, $p = 0.08$								
**Indication = non-ICH**								
Schmidt LE 2022	6	33	9	52	1.05	[0.41; 2.68]	4.2%	5.1%
Stevens VM 2021	2	16	5	16	0.40	[0.09; 1.77]	3.0%	2.4%
Sadek E 2024	15	59	49	265	1.37	[0.83; 2.28]	10.7%	11.0%
Koo SJ 2024	10	84	20	99	0.59	[0.29; 1.19]	11.0%	7.7%
**Common effect model**		192		432	0.93	[0.65; 1.33]	28.8%	.
**Random effects model**					0.90	[0.40; 2.03]	.	26.2%
Heterogeneity: $I^2 = 42\%$, $\tau^2 = 0.0928$, $p = 0.16$								
**Indication = mixed**								
Singer AJ 2023	8	50	9	50	0.89	[0.37; 2.12]	5.4%	5.7%
**Common effect model**		**730**		**931**	**0.84**	**[0.69; 1.01]**	**100.0%**	.
**Random effects model**					**0.82**	**[0.63; 1.07]**	.	**100.0%**

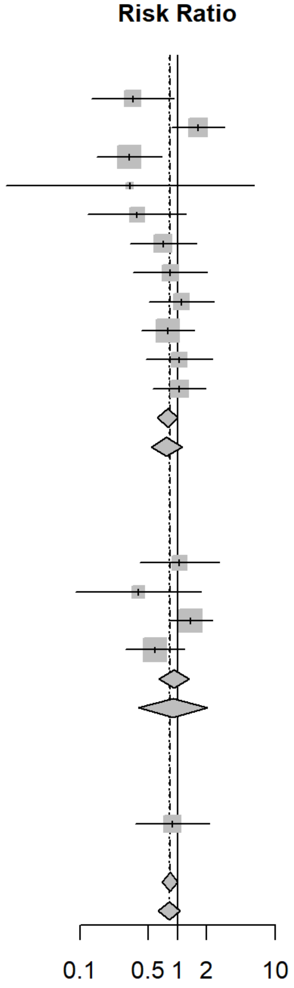

Heterogeneity: $I^2 = 34\%$, $\tau^2 = 0.0746$, $p = 0.09$
Test for subgroup differences (common effect): $\chi^2_2 = 0.53$, df = 2 ($p = 0.77$)
Test for subgroup differences (random effects): $\chi^2_2 = 0.29$, df = 2 ($p = 0.87$)

Figure 4s.

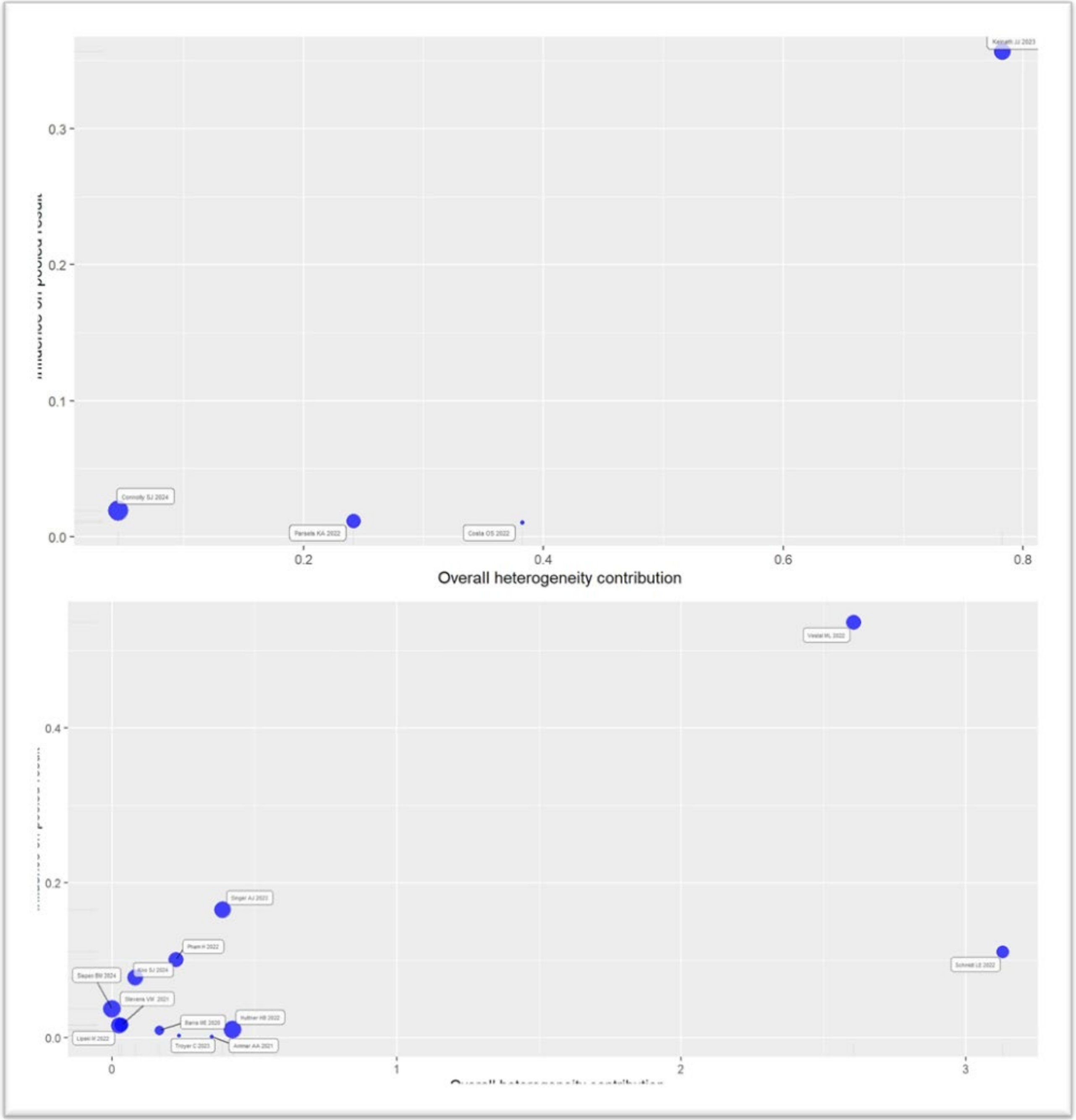
0.3
0.2
0.1
0.0
0.2
0.4
0.6
0.8
Overall heterogeneity contribution
0.4
0.2
0.0
0
1
2
3

Figure 5s

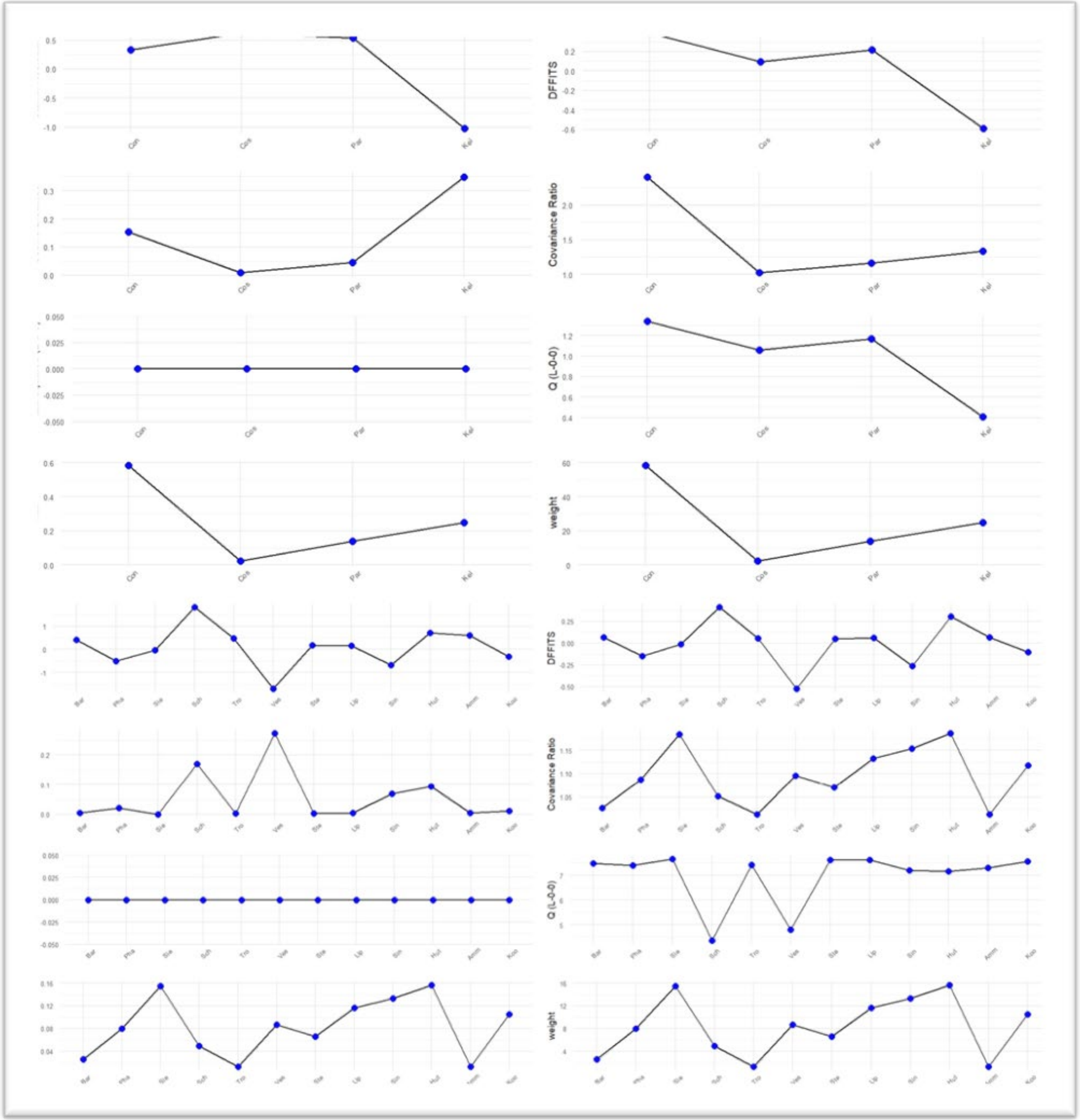

Figure 6s.

Study	Experimental Events	Experimental Total	Control Events	Control Total	RR	95%-CI	Weight (common)	Weight (random)
Indication = ICH								
Connolly SJ 2024	27	263	15	267	1.83	[1.00; 3.36]	57.5%	58.5%
Costa OS 2022	2	107	0	95	4.44	[0.22; 91.37]	0.0%	2.4%
Parsels KA 2022	7	26	3	26	2.33	[0.68; 8.05]	11.6%	14.1%
Common effect model		396		388	2.02	[1.17; 3.47]	69.1%	.
Random effects model					1.97	[1.16; 3.35]	.	74.9%
Heterogeneity: $I^2 = 0\%$, $\tau^2 = 0$, $p = 0.82$								
Indication = non-ICH								
Keinath JJ 2023	9	170	8	170	1.12	[0.44; 2.85]	30.9%	25.1%
Sutton SS 2023	.	85	.	170			0.0%	0.0%
Indication = mixed								
Cohen AT 2021	.	322	.	88			0.0%	0.0%
**Common effect model**		**973**		**816**	**1.74**	**[1.09; 2.77]**	**100.0%**	**.**
**Random effects model**					**1.71**	**[1.01; 2.89]**	**.**	**100.0%**

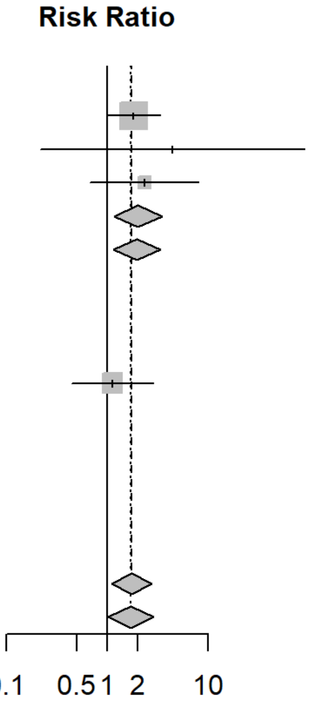

Heterogeneity: $I^2 = 0\%$, $\tau^2 = 0$, $p = 0.69$
Test for subgroup differences (common effect): $\chi^2_1 = 1.13$, df = 1 ($p = 0.29$)
Test for subgroup differences (random effects): $\chi^2_1 = 1.30$, df = 1 ($p = 0.25$)

Figure 7S

Study	Experimental Events	Experimental Total	Control Events	Control Total	Risk Ratio	RR	95%-CI	Weight (common)	Weight (random)
**Indication = ICH**									
Barra ME 2020	3	18	1	11		1.83	[0.22; 15.51]	2.2%	2.6%
Pham H 2022	4	47	6	62		0.88	[0.26; 2.94]	9.4%	8.0%
Siepen BM 2024	20	180	6	63		1.17	[0.49; 2.77]	16.1%	15.5%
Oh ES 2023	.	9	.	15				0.0%	0.0%
Troyer C 2023	2	31	0	15		2.46	[0.13; 48.19]	0.0%	1.3%
Vestal ML 2022	3	21	11	35		0.45	[0.14; 1.44]	14.9%	8.7%
Irizarry-Gatell VM	.	23	.	66				0.0%	0.0%
Lipski M 2022	5	23	8	47		1.28	[0.47; 3.47]	9.5%	11.6%
Huttner HB 2022	11	85	8	97		1.57	[0.66; 3.72]	13.5%	15.6%
Ammar AA 2021	2	28	0	16		2.89	[0.15; 56.72]	0.0%	1.3%
Milioglou I 2022	.	23	.	22				0.0%	0.0%
Common effect model		488		449		1.16	[0.76; 1.77]	65.7%	.
Random effects model						1.14	[0.77; 1.68]	.	64.7%
Heterogeneity: $I^2 = 0\%$, $\tau^2 = 0$, $p = 0.78$									
**Indication = non-ICH**									
Schmidt LE 2022	6	33	2	52		4.73	[1.01; 22.04]	2.8%	4.9%
Stevens VM 2021	4	16	3	16		1.33	[0.35; 5.03]	5.4%	6.6%
Sadek E 2024	.	59	.	265				0.0%	0.0%
Koo SJ 2024	6	84	7	99		1.01	[0.35; 2.89]	11.6%	10.5%
Common effect model		192		432		1.62	[0.81; 3.25]	19.9%	.
Random effects model						1.63	[0.24; 11.07]	.	22.1%
Heterogeneity: $I^2 = 26\%$, $\tau^2 = 0.1716$, $p = 0.26$									
**Indication = mixed**									
Singer AJ 2023	7	50	8	50		0.88	[0.34; 2.23]	14.5%	13.3%
**Common effect model**		**730**		**931**		**1.21**	**[0.87; 1.69]**	**100.0%**	**.**
**Random effects model**						**1.18**	**[0.86; 1.62]**	**.**	**100.0%**

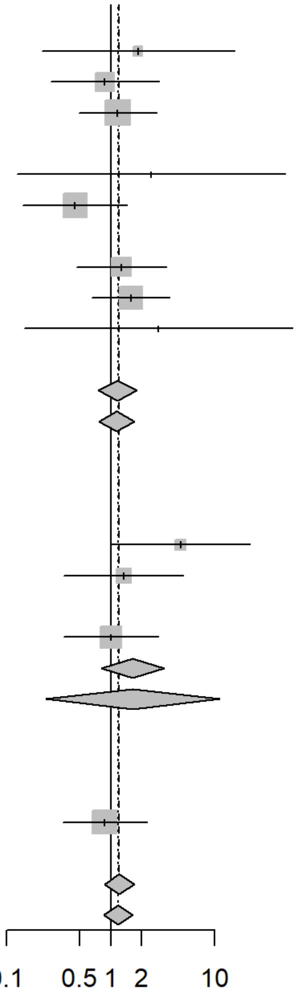

Heterogeneity: $I^2 = 0\%$, $\tau^2 = 0$, $p = 0.74$
Test for subgroup differences (common effect): $\chi^2_2 = 1.19$, df = 2 ($p = 0.55$)
Test for subgroup differences (random effects): $\chi^2_2 = 0.93$, df = 2 ($p = 0.63$)

Figure 8s.

Table 2s.

Study	Design	Mortality rate
ICH		
Connolly SJ 2024	RCT	0.23
Costa OS 2022	PSM	0.12
Barra ME 2020	Retrospective	0.37
Pham H 2022	Retrospective	0.27
Siepen BM 2024	Retrospective	0.09
Oh ES 2023	Retrospective	0.08
Troyer C 2023	Retrospective	0.20
Vestal ML 2022	Retrospective	0.36
Irizarry-Gatell VM	Retrospective	0.25
Lipski M 2022	Retrospective	0.29
Huttner HB 2022	Retrospective	0.19
Ammar AA 2021	Retrospective	0.39
Milioglou I 2022	Retrospective	0.47
Non-ICH		
Keinath JJ 2023	PSM	0.15
Sutton SS 2023	PSM	0.20
Schmidt LE 2022	Retrospective	0.18
Stevens VM 2021	Retrospective	0.22
Sadek E 2024	Retrospective	0.20
Koo SJ 2024	Retrospective	0.16
Mixed population		
Cohen AT 2021	PSM	0.19
Singer AJ 2023	Retrospective	0.17
